# Meals for Necessitous School Children during the Christmas Holidays

**Published:** 1910-12-24

**Authors:** A. F. London, H. Adler, J. Scott Lidgett, R. Melvill Beachcroft, John Kirk, E. A. H. Jay

**Affiliations:** Northampton


### Editor's Letter-Box.

### MEALS FOR NECESSITOUS SCHOOL CHILDREN DURING THE CHRISTMAS HOLIDAYS.

Sir,—The fact has to be admitted that there is in our midst a mass of underfed neglected childhood. This has been recognised by advantage having been taken of the "Provision of Meals Act, 1906," to provide meals for such of the children attending our London elementary schools as are necessitous, in order that they may be able to benefit from the education given. The Act, however, does not permit the Education Authority to supply such meals except during the school period. It follows that during the approaching Christmas holidays many thousands of children will sadly miss their accustomed daily meal.

In order to meet this want, the Education Committee, through its chairman, has invited the co-operation of the Ragged School Union, which is so largely engaged in helping the waifs and strays of the Metropolis. It is in these circumstances that we ask you, sir, to allow us to make an appeal through your columns.

For over twenty years before the Act came into operation the London Schools Dinner Association had aided greatly in providing meals for school children. The association is now a branch of the Ragged School Union working under a scheme of the Charity Commissioners, and its present funds will admit of a grant of between £100 and £200 towards the object now in view.

It is anticipated that if provision is now made for about half the necessitous children usually fed during school time, that will—having regard to help forthcoming from other private sources at such a time as Christmas—meet the actual needs. On this basis a further sum of about £1,000 must *at once* be secured if the necessary order is to be given. We feel sure that all who love children will wish that there should be no really hungry ones at this season of the year, and we therefore confidently ask for voluntary offerings towards the sum required. Subscribers may feel assured that the Ragged School Union, with the help of the local Associations of Care Committees, will take every care that there is proper discrimination in the selection of the children fed.

Contributions should be sent to Sir John Kirk, Ragged School Union, 32 John Street, Theobalds Road, W.C.

We are, Sir,
Your obedient servants,

Northampton, R. Melvill Beachcroft,

